# Beyond sum scores: a network analysis of dark triad traits and nonsuicidal self-injury in emerging adults

**DOI:** 10.3389/fpsyg.2026.1954866

**Published:** 2026-10-15

**Authors:** Weiman Yan, Siqi Ma, Tenglong Wang, Yajun Tian, Yulin Xu, Kewei Sun, Chaoqun Wang, Qingjun Guo

**Affiliations:** 1 School of Psychology, Shanghai Normal University, Shanghai, China; 2 Psychological Research Center, Xinjiang Taineng Psychological Counseling Institution, Xinjiang, China; 3 Mental Health Center, Chongqing Western Hospital, Chongqing, China; 4 Psychology Section, Secondary Sanatorium of Air Force Healthcare Center for Special Services, Hangzhou, China; 5 Department of Military Medical Psychology, Fourth Military Medical University, Xian, China; 6 Department of Endocrinology, The Third Affiliated Hospital of Naval Medical University, Shanghai, China

**Keywords:** bridge nodes, dark triad traits, emerging adults, network analysis, nonsuicidal self-injury

## Abstract

**Background:**

Previous studies have investigated the relationships between dark triad traits and nonsuicidal self-injury (NSSI) based on sum scores of corresponding scales. However, this practice has hampered the fine-grained understanding of how specific dark triad traits relate to NSSI at the item level. This network analysis study aimed to examine these relationships in a joint framework and identify potential intervention targets.

**Methods:**

Dark triad traits and NSSI were measured using the Dirty Dozen (DD) and the behavior subscale of the Adolescent Nonsuicidal Self-Injury Assessment Questionnaire (ANSAQ) among 1,950 emerging adults. A regularized partial correlation network was constructed, and expected influence and bridge expected influence were calculated for each node.

**Results:**

In addition to connections within each community, several cross-community edges were observed. The strongest direct edge between the Dark Triad Traits and NSSI was found between N4 “I tend to expect special favors from others” (narcissism) and B1 “Intentionally pinching myself” (NSSI), but this edge was small in magnitude. P1 “I tend to lack remorse” (psychopathy) and N1 “I tend to want others to admire me” (narcissism) had the highest expected influence values, whereas P4 “I tend to be cynical” (psychopathy) and P1 “I tend to lack remorse” (psychopathy) had the highest bridge expected influence values.

**Conclusion:**

These findings suggest that the item-level connections between dark triad traits and NSSI are generally weak, with the most prominent associations occurring within the Dark Triad Traits dimensions themselves. P1 and N1 appear to occupy central positions in the Dark Triad Traits network, while P4 and P1 mainly serve as bridges among the three dark traits traits rather than as direct links to NSSI. The direct association between narcissistic entitlement and intentional pinching may warrant further investigation, but its clinical significance remains unclear. Future research should examine whether these specific personality characteristics and self-injury behaviors can inform targeted prevention or intervention efforts among emerging adults with dark triad traits.

## 1 Introduction

Nonsuicidal self-injury (NSSI) refers to the direct and deliberate destruction of one’s own body tissue without any observable intent to die (Nock, 2010). NSSI is particularly common among adolescents and emerging adults, with a meta-analysis reporting lifetime prevalence rates of approximately 17.2 and 13.4%, respectively (Swannell et al., 2014). Critically, a history of NSSI significantly increases the risk of subsequent suicidal ideation and suicide attempts, making it one of the strongest predictors of future suicidal behavior (Hamza et al., 2012; Ribeiro et al., 2016). NSSI has therefore become a major public health concern that warrants sustained research attention, particularly during emerging adulthood, a developmental period characterized by distinct challenges and risk profiles.

Although NSSI typically begins in mid-adolescence, it does not necessarily remit with the end of adolescence (Plener et al., 2015). NSSI remains highly prevalent during emerging adulthood (18–25 years) (Reinhardt and Kökönyei, 2026; Cipriano et al., 2017), and some researchers have identified this period as a second peak in NSSI risk (Gandhi et al., 2018). Emerging adulthood represents a unique developmental stage marked by identity exploration, career decision-making, and adaptation to new social roles (Arnett, 2000), all of which are often accompanied by elevated stress and emotional distress (Arnett, 2007). Moreover, longitudinal evidence indicates that more than half of adolescents with a history of NSSI continue to engage in self-injury during early adulthood (Kiekens et al., 2023), while the likelihood of discontinuing NSSI is highest between the ages of 18 and 21 (Turner et al., 2022). Thus, emerging adulthood constitutes both a critical period for the persistence of NSSI and an important window for intervention. However, most existing research has focused on adolescents, and the fine-grained mechanisms underlying NSSI during emerging adulthood remain underexplored.

Previous research has consistently demonstrated that personality traits play an important role in NSSI (Brown, 2009; Kiekens et al., 2015). Among maladaptive personality traits, the Dark Triad Traits, which comprises Machiavellianism, narcissism, and psychopathy (Paulhus and Williams, 2002), has drawn increasing attention because of its robust associations with a wide range of maladaptive behaviors (Furnham et al., 2013). Emerging evidence suggests that the Dark Triad Traits is positively associated with NSSI (Wang and Jin, 2026). Notably, the contributions of its three components appear to differ: Machiavellianism and psychopathy have been consistently linked to NSSI, whereas findings regarding narcissism have been more mixed (Greitemeyer and Sagioglou, 2021; Finch et al., 2023). Critically, existing studies have almost exclusively relied on total or dimension scores to examine these associations, an approach that cannot reveal how specific personality characteristics relate to specific NSSI behaviors. Moreover, NSSI itself encompasses multiple methods, including cutting, burning, hitting, scratching, and head banging (Saraff and Pepper, 2014), and different methods may have distinct psychological correlates. To date, no study has examined the relationships between individual Dark Triad Traits items and specific NSSI behaviors within a joint framework.

Network analysis offers a novel approach to address this gap. Unlike traditional latent variable models, network analysis conceptualizes individual items as mutually interacting nodes and estimates the direct associations among them (Borsboom and Cramer, 2013). This approach makes it possible to investigate how specific personality characteristics are directly linked to specific NSSI behaviors, rather than assuming that all items merely reflect an underlying latent construct. Centrality indices can identify the most influential nodes within the network (Epskamp et al., 2018), while bridge centrality identifies nodes that connect different communities, offering insights into the mechanisms linking distinct psychological systems and revealing potential intervention targets (Jones et al., 2021; Borsboom, 2017). This approach has been fruitfully applied to both Dark Triad Traits and NSSI research. In the personality domain, Marcus et al. (2018) found that interpersonal manipulation and callousness were the most central traits in a network of dark personality characteristics, while Trahair et al. (2020) showed that Machiavellianism and psychopathy occupied more central positions than narcissism within the Dark Triad Traits network. In the NSSI domain, Reinhardt et al. (2025) found that severe scratching, cutting, and pinching were most strongly interconnected with other self-injury behaviors, and Xie et al. (2024) identified scratching and pinching as central NSSI nodes in a network of childhood maltreatment and NSSI. Despite these advances in each respective field, no study has yet integrated Dark Triad Traits items and NSSI behaviors within a single network framework.

To address this gap, the present study employed network analysis to examine the relationships between individual Dark Triad Traits items and specific NSSI behaviors among emerging adults with a history of NSSI. Specifically, we asked: (1) Which Dark Triad Traits items and NSSI behaviors occupy the most central positions within the network? (2) Which nodes serve as bridge nodes connecting the Dark Triad Traits and NSSI communities? (3) What are the specific pathways through which individual Dark Triad Traits items are associated with specific NSSI behaviors? By addressing these questions, we aimed to provide a more nuanced understanding of the associations between maladaptive personality traits and NSSI and to identify potential personality-based targets for precision intervention among emerging adults.

## 2 Methods

### 2.1 Participants and ethical approval

A cross-sectional survey design was adopted for the present study. Data were gathered through an online questionnaire hosted on Wenjuanxing^1^, a trusted and extensively utilized survey platform in China. The emerging adult sample was obtained from four Chinese provincial-level divisions (Shanghai, Xinjiang, Chongqing, and Zhejiang) using a non-probability convenience and snowball sampling strategy. The initial pool was accessed through the investigators’ personal networks, and subsequent respondents were invited via peer-to-peer sharing of the survey link. This non-probability sampling strategy may introduce selection bias and sample homogeneity, which could limit the generalizability of the findings to the broader emerging adult population. A total of 2,000 completed questionnaires were returned.

The eligibility requirements for participation were as follows: (1) being an emerging adult aged 18–25 years; (2) being free from any self-declared neurological or severe psychiatric condition; and (3) providing electronic informed consent on a voluntary basis. To protect data integrity, several attention check items (e.g., “Please select option 3 for this item”) were embedded throughout the questionnaire. Responses were flagged for removal if they met any of the following exclusion criteria: (1) incorrect responses to attention check items; (2) participant age falling outside the 18–25 bracket; or (3) clear indications of haphazard responding, such as uniform answering patterns or an unusually rapid completion time. After applying these quality control procedures, the final analytic sample consisted of 1,950 emerging adults.

Prior to beginning the survey, all individuals were informed in electronic form about the aims and nature of the investigation and were required to provide consent. The survey was administered anonymously, and participants retained the freedom to discontinue their involvement at any point without facing any repercussions. The entire research protocol was executed in strict compliance with the Declaration of Helsinki and received formal ethical clearance from the Ethics Committee of Shanghai Normal University.

### 2.2 Measures

#### 2.2.1 Dark triad traits

Dark Triad Traits were assessed using the Dirty Dozen (DD), a 12-item self-report instrument originally developed by Jonason and Webster (2010) and subsequently validated in Chinese populations by Geng et al. (2015). The scale measures three conceptually distinct but empirically correlated dimensions of the dark personality constellation: Machiavellianism (items 1–4), psychopathy (items 5–8), and narcissism (items 9–12). Each item is rated on a 7-point Likert scale ranging from 1 (“strongly disagree”) to 7 (“strongly agree”). Representative items include “I tend to manipulate others to get my way” (Machiavellianism), “I tend to be callous or insensitive” (Psychopathy), and “I tend to want others to admire me” (Narcissism). In the present sample, the internal consistency of the Machiavellianism, psychopathy, and narcissism dimensions (α = 0.858, 0.889, and 0.895, respectively) and the total DD scale (α = 0.784) were acceptable. The DD was selected rather than other Dark Triad instruments, such as the Short Dark Triad (SD3), for several reasons. First, the DD has been validated and used in Chinese populations and in previous item-level network studies of Dark Triad traits (Geng et al., 2015; Li et al., 2024; Wang et al., 2026). Second, its 12-item format is more concise than longer instruments such as the 27-item SD3, which reduces participant burden and limits the number of nodes in an item-level network, thereby helping to maintain network stability. Third, the DD captures the three core Dark Triad dimensions with four items per dimension, allowing us to examine specific Dark Triad characteristics within the network rather than only aggregate trait scores. Previous studies using the same instrument have likewise examined the Dark Triad Traits as three separate dimensions (Li et al., 2024; Wang et al., 2026). Moreover, this three-dimensional approach has been supported across different countries and populations, further supporting its applicability in the present study (Grabovac et al., 2025; Postigo et al., 2024).

#### 2.2.2 Nonsuicidal self-injury

Nonsuicidal self-injury was measured using the behavior subscale of the Adolescent Nonsuicidal Self-Injury Assessment Questionnaire (ANSAQ), revised for Chinese adolescents by Wan et al. (2018). This subscale comprises 12 items that capture the frequency of various self-harm methods without suicidal intent (e.g., “intentionally cutting myself,” “deliberately hitting myself”). Respondents indicated how often they had engaged in each behavior during the past 12 months, using a 5-point scale with the following anchors: 0 (“never”), 1 (“occasionally”), 2 (“sometimes”), 3 (“often”), and 4 (“always”) (Shi et al., 2026). Higher scores reflected greater engagement in NSSI acts. The instrument has demonstrated sound psychometric properties in prior research with Chinese youth. In the current investigation, the NSSI behavior subscale showed good internal consistency (Cronbach’s *α* = 0.959).

### 2.3 Statistical analysis

#### 2.3.1 Assessment of common method variance

Given that all variables were assessed using self-report measures at a single time point, Harman’s single-factor test was conducted as a preliminary assessment of common method variance. Principal component analysis of all 24 items showed that the first factor accounted for 38.490% of the total variance, which was below the commonly used 40% threshold. This result did not indicate that a single common factor accounted for a substantial proportion of the variance in the present data. However, Harman’s single-factor test provides only a limited diagnostic of CMV and cannot definitively rule out common method bias. Therefore, the present result should be interpreted cautiously.

#### 2.3.2 Network estimation and visualization

An Extended Bayesian Information Criterion (EBIC) graphical least absolute shrinkage and selection operator (LASSO) network model was estimated (Epskamp and Fried, 2018; Friedman et al., 2008). This study predefined four communities of nodes based on the validated structure of the instruments: Machiavellianism (M1–M4), psychopathy (P1–P4), narcissism (N1–N4), and NSSI (B1–B12). Each edge in the network corresponds to the partial correlation between a pair of nodes, with all other nodes in the model statistically controlled. Considering the ordinal response format of the Likert-type items, the network was constructed using nonparametric Spearman correlations as the input (Epskamp and Fried, 2018). The LASSO regularization procedure was then employed to shrink negligible or trivial partial correlations to exactly zero, yielding a sparse and interpretable network structure (Friedman et al., 2008). The EBIC tuning hyperparameter γ was specified at 0.5, a setting that achieves a balanced trade-off between detecting genuine edges and excluding spurious ones (Foygel and Drton, 2010).

The resulting network was plotted using the qgraph package in R (Epskamp et al., 2012). In the visual representation, nodes with stronger interconnections are positioned in closer proximity to one another, whereas nodes with weak or absent connections are located farther apart. Positive partial correlations are denoted by blue edges, and negative partial correlations are denoted by red edges. The width of each edge is proportional to the magnitude of the corresponding association, such that thicker lines reflect larger edge weights.

#### 2.3.3 Centrality and bridge centrality analysis

Expected influence (EI) was computed for each node as the sum of raw edge weights (both positive and negative) connecting that node to all other nodes in the network (Robinaugh et al., 2016). Higher EI values indicate greater centrality in the overall network.

In addition to overall centrality, we examined cross-community connectivity. To identify nodes that connect different communities, bridge expected influence (BEI) was calculated using the networktools package (Jones et al., 2021). BEI represents the sum of edge weights connecting a given node to all nodes outside its own community. Because positive and negative cross-community edges can cancel each other, bridge strength (the sum of absolute cross-community edge weights) was also computed as a sensitivity index.

Based on the EI and BEI indices described above, central and bridge nodes were identified using a prespecified rule. First, nodes with the highest EI or BEI values were treated as candidate nodes. Second, bootstrap difference tests (1,000 samples) were used to compare each candidate’s EI or BEI with those of all other nodes (Epskamp et al., 2018). A node was reported as central only if its EI was significantly higher than at least 65% of the remaining nodes, and as a bridge node only if its BEI was significantly higher than at least 90% of the remaining nodes. These thresholds were set *a priori* to reduce the risk of interpreting small numerical differences as meaningful (Fried et al., 2017).

#### 2.3.4 Network accuracy and stability

To evaluate the robustness of the above results, two bootstrap-based procedures were implemented with 1,000 iterations each using the bootnet package in R (Epskamp et al., 2018). First, nonparametric bootstrapping was conducted to construct 95% confidence intervals (CIs) around each edge weight; narrower CIs reflect more precise edge weight estimates. Second, the stability of the centrality indices (EI and BEI) was examined through a case-dropping subset bootstrap, in which the proportion of cases dropped ranged from 30 to 75% in steps of 5%. The correlation stability (CS) coefficient was calculated using a correlation threshold of 0.70, representing the maximum proportion of the sample that can be dropped while retaining a correlation of at least 0.70 between the original centrality values and those derived from the reduced sample, with 95% probability (Epskamp et al., 2018). The stability of bridge strength was evaluated using the same case-dropping procedure. Following established guidelines, CS coefficients exceeding 0.50 are considered indicative of strong stability, whereas values below 0.25 raise substantial concerns about the reliability of the centrality ordering (Epskamp et al., 2018). In addition, bootstrap difference tests for edge weights were conducted, with a statistically significant difference inferred when the bootstrapped 95% CIs for the difference did not contain zero (Epskamp et al., 2018).

## 3 Results

The mean age of the participants was 18.98 years (SD = 1.02, range = 18–25 years). The sample included 775 males (39.74%) and 1,175 females (60.26%). Among them, 343 (17.59%) were only children, and 1,607 (82.41%) had siblings; 236 (12.10%) were from single-parent families, and 1,714 (87.90%) were from non-single-parent families. Detailed demographic characteristics are presented in Table 1. With regard to NSSI, 22.359% of participants reported engaging in at least one NSSI behavior during the past 12 months. Table 2 shows the mean and standard deviation of each variable in the current network analysis.

**Table 1:** Demographic characteristics of the participants (*N* = 1,950).

Variable	Category	*n* (%)
Gender	Male	775 (39.744%)
	Female	1,175 (60.256%)
Age(years)	18	774 (39.692%)
	19	615 (31.538%)
	20	427 (21.897%)
	21	105 (5.38%)
	22	18 (0.923%)
	23	6 (0.308%)
	24	3 (0.154%)
	25	2 (0.103%)
Grade	Freshman	764 (39.179%)
	Sophomore	587 (30.103%)
	Junior	589 (30.205%)
	Senior	10 (0.513%)
Only child	No	1,607 (82.410%)
	Yes	343 (17.59%)
Single-parent family	No	1,714 (87.897%)
	Yes	236 (12.103%)
Marital Status	Divorced	12 (0.615%)
	Single	1,929 (98.923%)
	Married	9 (0.462%)
Monthly per capita household income (RMB)	Below 3,000	724 (37.128%)
	3,000–5,000	767 (39.333%)
	5,000–10,000	367 (18.821%)
	Above 10,000	92 (4.718%)

**Table 2:** Item labels for the dirty dozen (DD) and the adolescent nonsuicidal self-injury assessment questionnaire behavior subscale (ANSAQ).

Items	Abb	M	SD	EI	BEI
NSSI					
Intentionally pinching myself.	B1	0.235	0.625	0.970	0.126
Intentionally scratching myself.	B2	0.183	0.575	0.976	0.005
Intentionally banging my head against hard objects (walls, trees, etc.).	B3	0.165	0.535	1.044	0.009
Intentionally hitting walls, tables, windows, floors, etc., with my fist.	B4	0.188	0.557	0.896	0.058
Intentionally hurting myself with my fist, palm, or a harder object.	B5	0.151	0.520	1.059	0.010
Intentionally biting myself.	B6	0.154	0.526	0.989	0.017
Intentionally pulling out my own hair.	B7	0.151	0.555	0.781	0.007
Intentionally poking or stabbing myself (e.g., needles, staples, pen tips, etc.).	B8	0.109	0.440	1.064	0.001
Intentionally cutting myself (e.g., blades, glass, etc.).	B9	0.112	0.457	0.950	0.033
Intentionally burning or scalding myself (e.g., with cigarette butts, boiling water, lighters, or matches).	B10	0.083	0.399	1.010	−0.027
Deliberately rubbing my skin with an object to cause bleeding or bruising.	B11	0.091	0.410	0.965	0.000
Intentionally marking my skin with letters or symbols (excluding tattoos).	B12	0.085	0.394	0.894	−0.022
DD					
I tend to manipulate others to get my way.	M1	1.807	0.694	0.825	0.348
I have used deceit or lied to get my way.	M2	1.712	0.733	0.834	0.370
I have use flattery to get my way.	M3	1.717	0.802	0.920	0.463
I tend to exploit others towards my own end.	M4	1.879	0.877	0.892	0.544
I tend to lack remorse.	P1	1.758	0.898	1.110	0.722
I tend to be unconcerned with the morality of my actions.	P2	1.636	0.707	0.943	0.504
I tend to be callous or insensitive.	P3	1.759	0.731	1.024	0.607
I tend to be cynical.	P4	1.953	1.043	0.940	0.780
I tend to want others to admire me.	N1	4.373	2.397	1.084	0.546
I tend to want others to pay attention to me.	N2	3.329	1.904	0.949	0.370
I tend to seek prestige or status.	N3	1.838	0.980	0.952	0.530
I tend to expect special favors from others.	N4	3.915	2.289	0.899	0.442

NSSI, nonsuicidal self-injury; DD, Dirty Dozen; M, mean; SD, standard deviation.

### 3.1 Network structure

Figure 1 shows the Dark Triad Traits–NSSI network. Based on the theoretical four-dimensional structure, four communities were defined *a priori*: Machiavellianism, Psychopathy, Narcissism, and NSSI. In the network with 24 nodes, 135 out of 276 possible edges were non-zero, including 8 negative edges and 127 positive edges. Overall, 48.913% of all possible edges were non-zero, indicating a moderate level of overall connectivity in the network.

**Figure 1:**
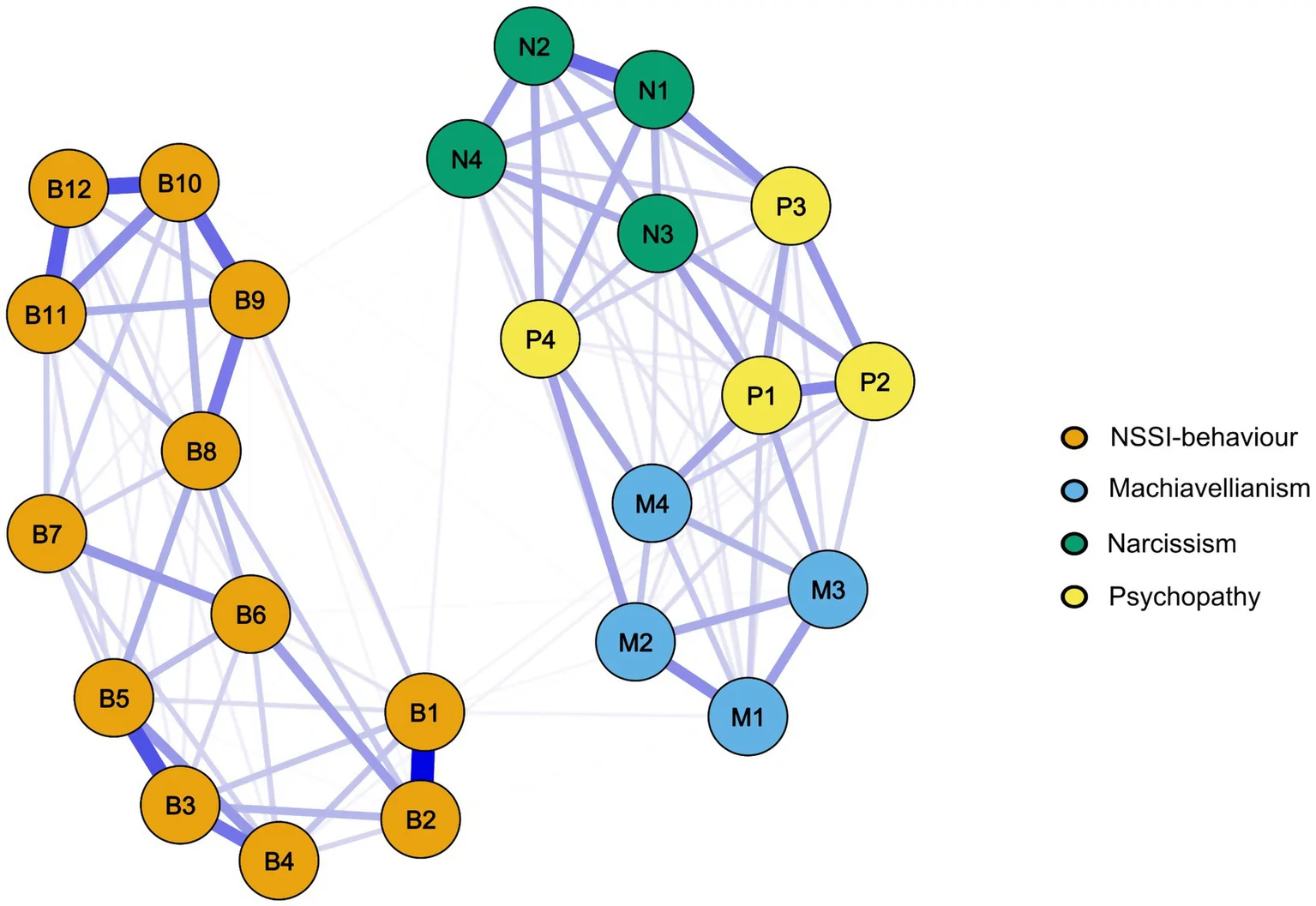
Network model of dark triad traits and NSSI. The blue line represents a positive partial correlation, and the red line represents a negative partial correlation. A thicker line and more saturated color indicate a larger partial correlation coefficient. Nodes are grouped into four communities: Machiavellianism (M1–M4), psychopathy (P1–P4), narcissism (N1–N4), and NSSI (B1–B12). Specific item labels are shown in Table 2.

Within the Machiavellianism community, the strongest edge was between M1 “I tend to manipulate others to get my way” and M2 “I have used deceit or lied to get my way” (weight = 0.205). Within the psychopathy community, the strongest edge was between P1 “I tend to lack remorse” and P2 “I tend to be unconcerned with the morality of my actions” (weight = 0.213). Within the narcissism community, the strongest edge was between N1 “I tend to want others to admire me” and N2 “I tend to want others to pay attention to me” (weight = 0.265). Within the NSSI community, the strongest edge was between B1 “Intentionally pinching myself” and B2 “Intentionally scratching myself” (weight = 0.447), followed by B3 “Intentionally banging my head against hard objects” and B5 “Intentionally hurting myself with my fist, palm, or a harder object” (weight = 0.309), and B10 “Intentionally burning or scalding myself” and B12 “Intentionally marking my skin with letters or symbols” (weight = 0.306).

The cross-community edges were generally weaker than the within-community edges. Among the three Dark Triad Traits dimensions, several relatively moderate edges were identified. Specifically, P3 “I tend to be callous or insensitive” (psychopathy) was positively associated with N1 “I tend to want others to admire me” (narcissism) (weight = 0.194); P4 “I tend to be cynical” (psychopathy) was positively associated with M2 “I have used deceit or lied to get my way” (Machiavellianism) (weight = 0.163); P1 “I tend to lack remorse” (psychopathy) was positively associated with M4 “I tend to exploit others towards my own end” (Machiavellianism) (weight = 0.155); and P2 “I tend to be unconcerned with the morality of my actions” (psychopathy) was positively associated with N3 “I tend to seek prestige or status” (narcissism) (weight = 0.154).

Cross-community edges involving NSSI were considerably smaller in absolute magnitude than within-community edges and were generally very weak. The largest was between N4 “I tend to expect special favors from others” (narcissism) and B1 “Intentionally pinching myself” (NSSI) (weight = 0.035), followed by M1 “I tend to manipulate others to get my way” (Machiavellianism) and B1 “Intentionally pinching myself” (NSSI) (weight = 0.034), and P2 “I tend to be unconcerned with the morality of my actions” (psychopathy) and B4 “Intentionally hitting walls, tables, windows, floors, etc., with my fist” (NSSI) (weight = 0.031). These edge weights were very small in magnitude, indicating that the direct connections between individual Dark Triad items and NSSI behaviors were minimal. Given the very small magnitude of these cross-community edges, the centrality and bridge-centrality results should be interpreted with caution.

Most edges in the network were positive, with only a few negative edges of small magnitude, such as B1 “Intentionally pinching myself” and B10 “Intentionally burning or scalding myself” (weight = −0.031), M1 “I tend to manipulate others to get my way” and B12 “Intentionally marking my skin with letters or symbols” (weight = −0.014), and N2 “I tend to want others to pay attention to me” and B10 “Intentionally burning or scalding myself” (weight = −0.009).

All edge weights of the Dark Triad Traits–NSSI network are provided in Supplementary Table S1. The bootstrapped 95% CIs for all edge weights are shown in Supplementary Figure S1. Full-precision edge estimates, bootstrap means, 95% CIs, and inclusion frequencies are reported in Supplementary Table S2. The bootstrapped difference test for edge weights is presented in Supplementary Figure S2.

### 3.2 Central nodes

The EI indices of the nodes are shown in Figure 2. P1 “I tend to lack remorse” (psychopathy) and N1 “I tend to want others to admire me” (narcissism) had the highest EI values (1.110 and 1.084, respectively). The bootstrapped difference test showed that the EIs of P1 “I tend to lack remorse” and N1 “I tend to want others to admire me” were significantly higher than approximately 65–70% of the other node EIs; no significant difference was observed between these two nodes (Supplementary Figure S3).

**Figure 2:**
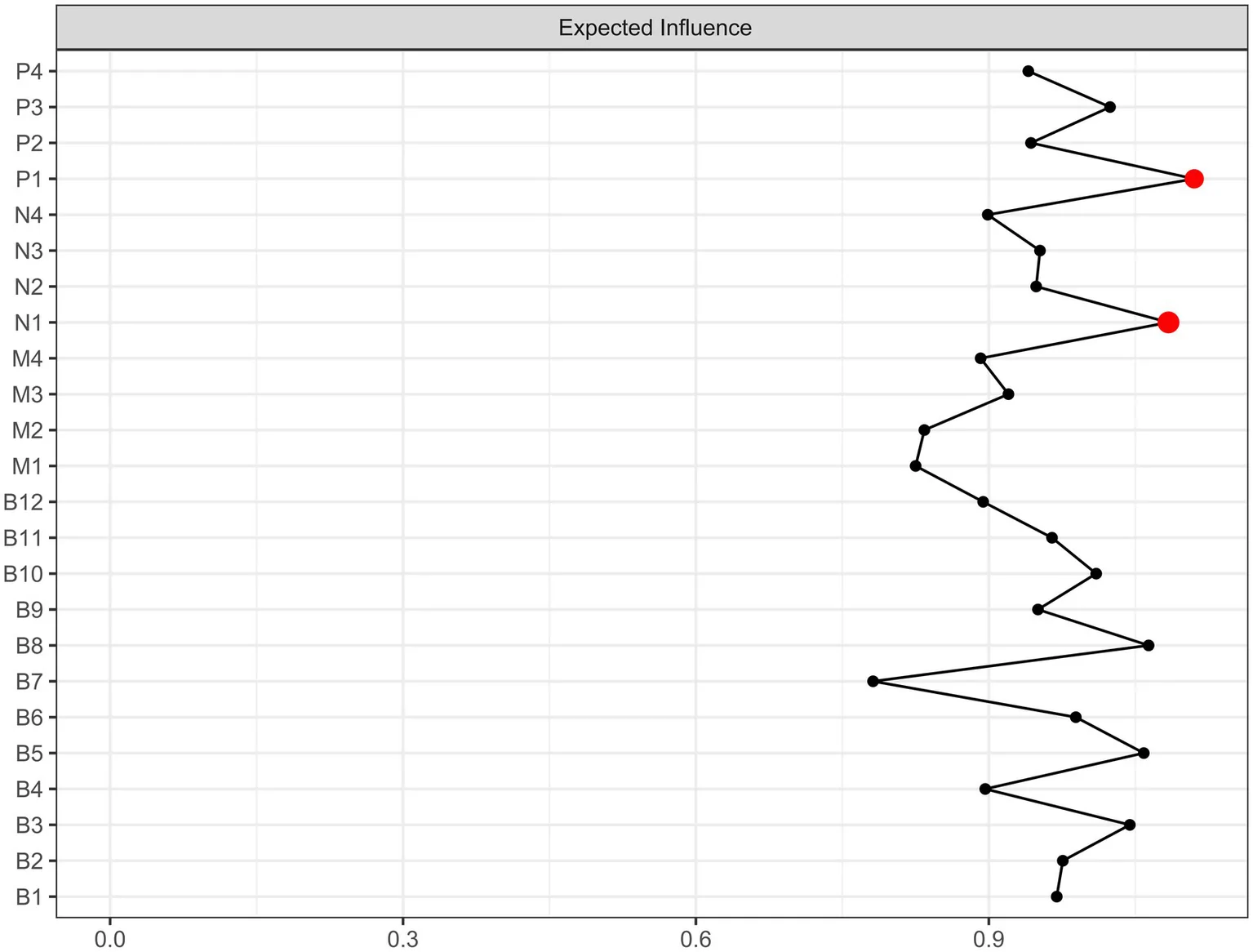
The expected influence of each node in the present network (raw values). Red nodes indicate the nodes with the highest expected influence values, representing the most central nodes in the network. Nodes are grouped into four communities: Machiavellianism (M1–M4), psychopathy (P1–P4), narcissism (N1–N4), and NSSI (B1–B12). Specific item labels are shown in Table 2.

### 3.3 Bridge nodes

The BEI of each node is presented in Figure 3A. P4 “I tend to be cynical” (psychopathy) and P1 “I tend to lack remorse” (psychopathy) had the highest BEI values (0.780 and 0.722, respectively), with no significant difference between them. Their BEIs were significantly higher than approximately 91–96% of the other nodes (see Supplementary Figure S4). P3 “I tend to be callous or insensitive” (psychopathy) also showed a relatively high BEI (0.607), though it was significantly lower than P4 “I tend to be cynical” (psychopathy). These BEI values should be interpreted alongside the very weak direct Dark Triad–NSSI edges observed in the present network. As reported above, the largest cross-community edges involving NSSI were approximately 0.03, indicating that the direct associations between individual Dark Triad items and NSSI behaviors were minimal. Bridge centrality therefore reflects the extent to which a node connects communities through its overall pattern of edges and should not be equated with the strength of direct Dark Triad–NSSI associations.

**Figure 3:**
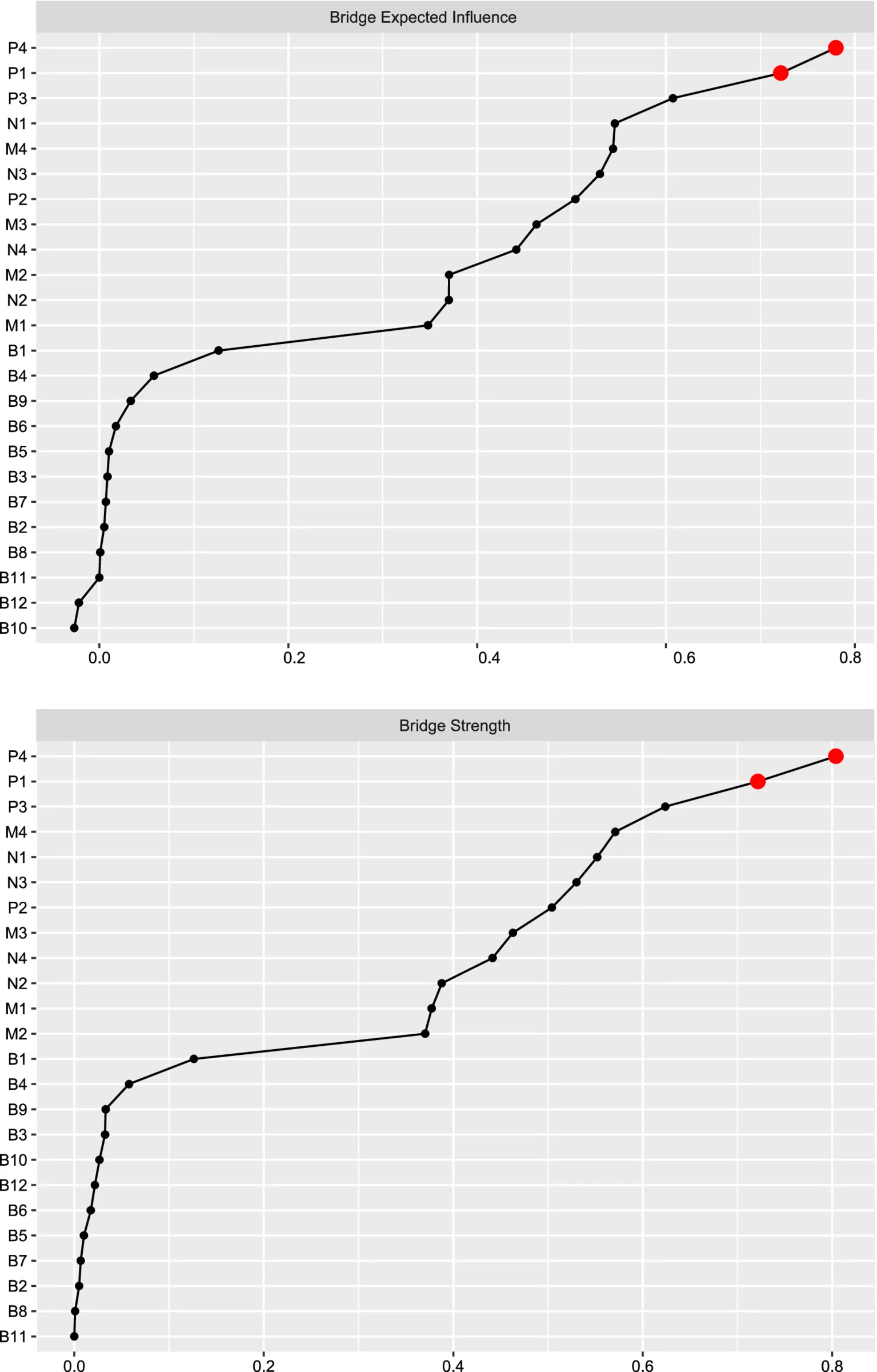
**(A)** Bridge expected influence and **(B)** bridge strength of each node in the present network (raw values). Red nodes indicate the nodes with the highest values, representing the most important bridge nodes. Nodes are grouped into four communities: Machiavellianism (M1–M4), psychopathy (P1–P4), narcissism (N1–N4), and NSSI (B1–B12). Specific item labels are shown in Table 2.

In terms of their connections, P4 “I tend to be cynical” (psychopathy) showed its strongest cross-community edges with M2 “I have used deceit or lied to get my way” (Machiavellianism) and N1 “I tend to want others to admire me” (narcissism), whereas it’s direct edges to NSSI items were small. Similarly, P1 “I tend to lack remorse” (psychopathy) showed its strongest cross-community edges within the Dark Triad Traits dimensions, with weak direct edges to NSSI. In contrast, the nodes with the strongest direct connections to NSSI were N4 “I tend to expect special favors from others” (narcissism) and B1 “Intentionally pinching myself” (NSSI), but their BEI values were lower than those of P4 “I tend to be cynical” (psychopathy) and P1 “I tend to lack remorse” (psychopathy).

As a sensitivity check, bridge strength was also computed and is presented in Figure 3B. The pattern of bridge strength was consistent with the BEI results, with P4 “I tend to be cynical” (psychopathy) and P1 “I tend to lack remorse” (psychopathy) remaining the highest, confirming the robustness of these findings.

### 3.4 Network accuracy and stability

Figure 4 displays the CS coefficients derived from the case-dropping bootstrap procedure. The case-dropping proportions ranged from 30 to 75% in steps of 5%, and the correlation threshold was set at 0.70. The CS-coefficient was 0.594 for EI. For BEI, the CS-coefficient was 0.75, which was the largest case-dropping proportion tested and therefore should be interpreted as at least 0.75 (boundary-limited). The bridge strength sensitivity analysis also showed a CS coefficient of 0.75, which should likewise be interpreted as boundary-limited. Both CS-coefficients exceeded the recommended threshold of 0.50, indicating that the centrality indices and bridge centrality indices demonstrated good stability.

**Figure 4:**
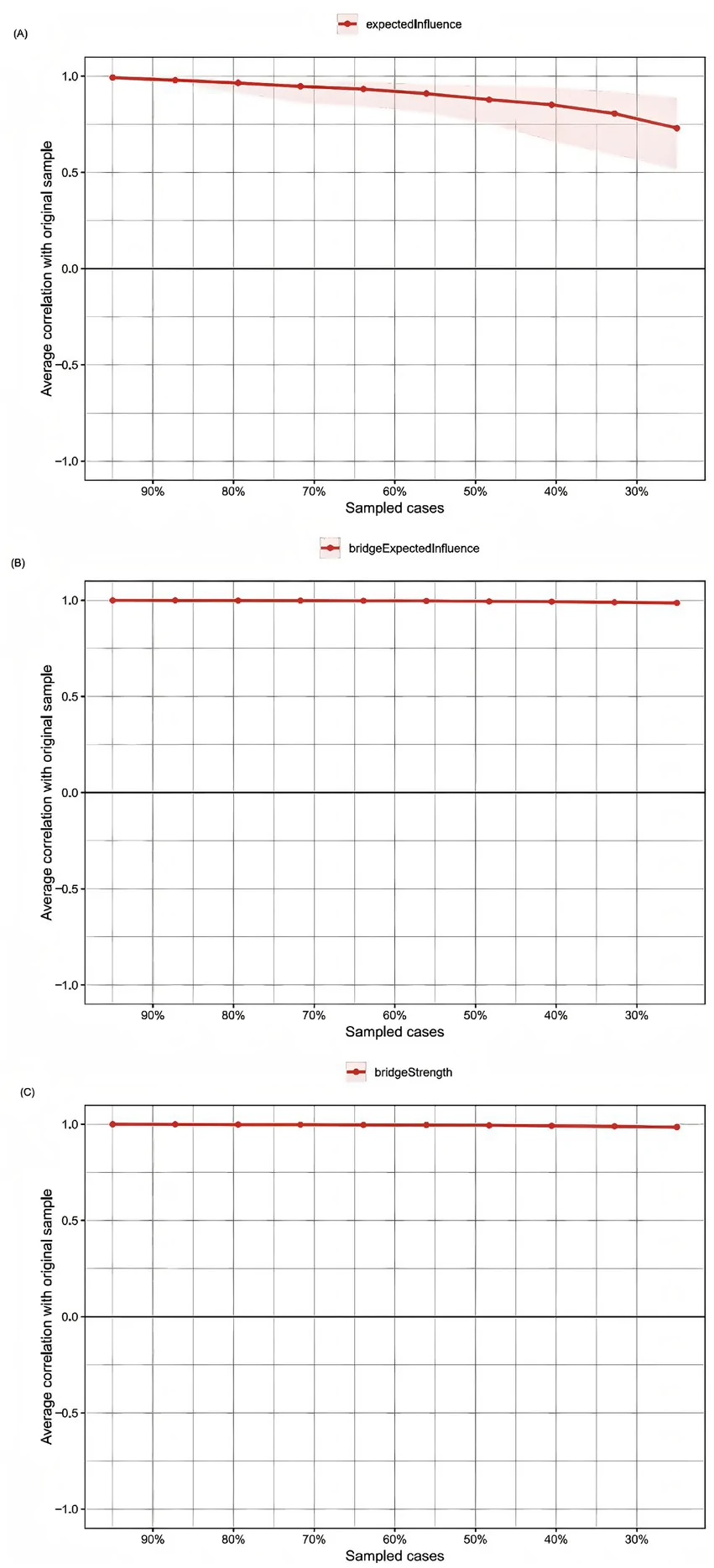
Stability of node expected influence, bridge expected influence, and bridge strength in the network. **(A)** Stability of expected influence. **(B)** Stability of bridge expected influence. **(C)** Stability of bridge strength. The red bar represents the average correlation between node centrality indices in the full sample and subsample, with the red area depicting the 2.5th quantile to the 97.5th quantile.

## 4 Discussion

To the best of our knowledge, this is the first study to apply network analysis to investigate item-level associations between specific Dark Triad Traits and individual NSSI behaviors within a unified framework. Adopting this approach, we estimated a network comprising four communities: Machiavellianism, psychopathy, narcissism, and NSSI. Consistent with prior network studies, the strongest edges were observed within communities, whereas cross-community edges were relatively weak and limited in number. These results suggest that specific Dark Triad Traits characteristics and NSSI behaviors are connected through particular pathways at the item level. In addition to the overall network structure, we examined node centrality and identified several nodes that occupied potentially influential positions within the network as either central or bridge nodes. From a network perspective, these findings offer a more fine-grained understanding of how distinct dark personality characteristics relate to specific forms of NSSI and suggest potential targets for future prevention and intervention research.

The analysis further showed that the strongest edges appeared within communities rather than between them. This is consistent with prior network studies in which variables belonging to the same construct tend to be more strongly interconnected than variables from different constructs (Li et al., 2024; Wang et al., 2026; Grabovac et al., 2025). Within the Dark Triad Traits communities, the strongest edges were between N1 “I tend to want others to admire me” (narcissism) and N2 “I tend to want others to pay attention to me” (narcissism), between P1 “I tend to lack remorse” (psychopathy) and P2 “I tend to be unconcerned with the morality of my actions” (psychopathy), and between M1 “I tend to manipulate others to get my way” (Machiavellianism) and M2 “I have used deceit or lied to get my way” (Machiavellianism). These edges are conceptually expected: admiration seeking and attention seeking represent closely related facets of grandiose narcissism (Back et al., 2013), whereas lack of remorse and moral unconcern are core affective features of psychopathy (Patrick et al., 2009). The M1–M2 edge, although somewhat weaker, reflects the manipulative and deceptive nature of Machiavellianism (Jones and Paulhus, 2014). Within the NSSI community, the strongest edge was between B1 “Intentionally pinching myself” (NSSI) and B2 “Intentionally scratching myself” (NSSI), which is consistent with prior studies showing that these two behaviors are strongly correlated and share similar procedural features (Reinhardt et al., 2025; Xie et al., 2024; He et al., 2026). Both behaviors can be performed without tools, produce similar sensory feedback, and typically result in mild tissue damage (Wan et al., 2018; Xie et al., 2024). Altogether, it is unsurprising that the strongest edges existed within each community, as items belonging to the same psychological construct are theoretically expected to interact closely with one another.

Beyond the within-community edges, several cross-community associations were identified, although they were generally small in magnitude. Among the three Dark Triad Traits dimensions, relatively moderate edges were observed. For example, P3 “I tend to be callous or insensitive” (psychopathy) was positively associated with N1 “I tend to want others to admire me” (narcissism); P4 “I tend to be cynical” (psychopathy) was positively associated with M2 “I have used deceit or lied to get my way” (Machiavellianism); P1 “I tend to lack remorse” (psychopathy) was positively associated with M4 “I tend to exploit others towards my own end” (Machiavellianism); and P2 “I tend to be unconcerned with the morality of my actions” (psychopathy) was positively associated with N3 “I tend to seek prestige or status” (narcissism). These edges are consistent with the view that Machiavellianism, psychopathy, and narcissism are related yet distinguishable constructs with overlapping interpersonal features (Marcus et al., 2018; Trahair et al., 2020; Grabovac et al., 2025). Regarding cross-community edges involving NSSI, the largest was between N4 “I tend to expect special favors from others” (narcissism) and B1 “Intentionally pinching myself” (NSSI), followed by the edge between M1 “I tend to manipulate others to get my way” (Machiavellianism) and B1 “Intentionally pinching myself” (NSSI), and the edge between P2 “I tend to be unconcerned with the morality of my actions” (psychopathy) and B4 “Intentionally hitting walls, tables, windows, floors, etc., with my fist” (NSSI). These direct cross-community edges were considerably weaker than the within-community edges, suggesting that the item-level associations between Dark Triad Traits and NSSI behaviors are generally weak. This pattern is broadly consistent with prior evidence that associations between broad dark personality traits and NSSI at the aggregate level are often modest (Li et al., 2024; Wang et al., 2026). Since no prior studies have examined the relationships between individual Dark Triad Traits items and specific NSSI behaviors, the present study provides preliminary insight into this issue, which warrants further investigation.

Consistent with the expectation that some nodes would be more influential than others, P1 “I tend to lack remorse” (psychopathy) and N1 “I tend to want others to admire me” (narcissism) were identified as the central nodes with the highest expected influence values. This finding aligns with previous network research in which lack of remorse and callousness have shown relatively high centrality within dark personality networks (Marcus et al., 2018; Trupp et al., 2021; West et al., 2023), and in which narcissistic admiration has been identified as a central feature of narcissism (Back et al., 2013). These characteristics have also been associated with emotional difficulties in prior non-network research; for example, Gómez-Leal et al. (2019) found that callous affect, a psychopathy facet closely related to lack of remorse, was associated with depressive symptoms, and Gogola et al. (2021) reported that all three Dark Triad Traits components were positively associated with depressive symptoms. However, it is important to note that their high centrality primarily reflected connections within the Dark Triad Traits communities rather than direct associations with NSSI. In other words, P1 “I tend to lack remorse” and N1 “I tend to want others to admire me” were central to the internal organization of the Dark Triad Traits, but they did not function as direct links to NSSI behaviors.

The bridge centrality index analysis identified P4 “I tend to be cynical” (psychopathy) and P1 “I tend to lack remorse” (psychopathy) as the nodes with the highest bridge expected influence values. Bridge nodes that connect different communities are crucial for understanding how one construct may influence another (Jones et al., 2021). However, further inspection of their connections indicated that their bridging roles mainly reflected connections among the three Dark Triad Traits dimensions rather than direct links to NSSI. Specifically, P4 “I tend to be cynical” showed its strongest cross-community edges with M2 “I have used deceit or lied to get my way” and N1 “I tend to want others to admire me,” whereas P1 “I tend to lack remorse” showed its strongest cross-community edges within the Dark Triad Traits dimensions, with only weak direct edges to NSSI. Notably, the nodes with the strongest direct connections to NSSI were N4 “I tend to expect special favors from others” (narcissism) and B1 “Intentionally pinching myself” (NSSI), but their bridge expected influence values were lower than those of P4 and P1. This pattern is consistent with prior network research suggesting that certain dark personality facets may serve primarily as bridges to other personality domains rather than as direct correlates of the outcome (McIlvenna et al., 2025; Grabovac et al., 2025). Collectively, these results indicate that the most prominent bridging roles in this network occur among the Dark Triad Traits dimensions themselves, rather than between Dark Triad Traits and NSSI.

The present findings have several theoretical and clinical implications. Theoretically, this study demonstrates that an item-level network approach can reveal which specific Dark Triad Traits characteristics are interconnected and which specific NSSI behaviors tend to co-occur. These findings shed light on the possible fine-grained links between dark personality characteristics and NSSI behaviors. However, because the direct cross-construct associations were weak, the findings should not be interpreted as supporting strong personality-to-NSSI pathways. Clinically, network theory holds that interventions targeting central nodes may disrupt the overall network, while targeting bridge nodes may reduce the connectivity between co-occurring constructs (Borsboom, 2017; Jones et al., 2021). In the present study, P1 “I tend to lack remorse” (psychopathy) and N1 “I tend to want others to admire me” (narcissism) may deserve attention as potential indicators of risk within the Dark Triad Traits network, whereas P4 “I tend to be cynical” (psychopathy) and P1 “I tend to lack remorse” (psychopathy) may be relevant to understanding the connections among the three Dark Triad Traits dimensions. However, because the direct associations with NSSI were weak, these nodes should not be regarded as established intervention targets. Future longitudinal and intervention studies are needed to determine whether changes in these specific characteristics are associated with changes in NSSI.

Several limitations should be noted. First, the cross-sectional design precludes causal inference (Epskamp et al., 2018), and longitudinal network studies are needed to examine temporal dynamics among the key nodes. Second, the reliance on self-report measures may have introduced social desirability bias, recall bias, and common method variance. Although Harman’s single-factor test did not indicate that a single factor accounted for the majority of the variance, this test provides only a limited diagnostic of common method variance and cannot definitively rule out residual method-related bias. Fourth, statistical centrality does not automatically translate into clinical utility (Fried et al., 2017; Mullarkey et al., 2019). Fifth, although the items were theoretically distinct, future research should conduct redundancy analyses to rule out empirical overlap (Fried and Cramer, 2017). Sixth, the network structure is specific to the scales used in this study, and replication with alternative measures is necessary. Seventh, participants were recruited using a non-probability convenience and snowball sampling strategy. This approach may have introduced selection bias and sample homogeneity, which could have influenced the estimated network edges (Erickson, 1979) and may limit the generalizability of the findings to the broader emerging adult population. Future studies using probability-based or more representative sampling methods are therefore warranted. Eighth, the exclusion of participants with self-reported severe psychiatric conditions may have restricted the variability of NSSI and related characteristics. Given the high comorbidity between NSSI and several psychiatric conditions, this range restriction may have attenuated some Dark Triad Traits–NSSI associations and influenced the estimated bridge centrality of nodes connecting the two communities. Therefore, the observed network structure may not generalize to clinical populations with NSSI, and future studies should replicate these findings in clinical samples. Finally, the four-community structure was defined based on the validated dimensions of the instruments rather than through data-driven community detection methods such as exploratory graph analysis. Future research may apply such methods to further examine the robustness of this community structure.

## 5 Conclusion

This study applied network analysis to investigate the relationships between Dark Triad Traits and NSSI among emerging adults at the item level. The most central nodes were P1 (“I tend to lack remorse”) from the psychopathy dimension and N1 (“I tend to want others to admire me”) from the narcissism dimension. The highest bridge expected influence values were observed for P4 (“I tend to be cynical”) and P1 (“I tend to lack remorse”), both from the psychopathy dimension; however, these bridging roles mainly reflected connections among the three Dark Triad Traits dimensions rather than direct links to NSSI. The strongest direct edge between the Dark Triad Traits and NSSI was found between N4 (“I tend to expect special favors from others”) and B1 (“Intentionally pinching myself”), but this edge was small in magnitude. Overall, these findings indicate that the item-level associations between Dark Triad Traits and NSSI are generally weak. Although the cross-sectional design precludes causal inference, the results highlight the need for further longitudinal and intervention research to examine whether these specific characteristics and behaviors have practical utility for understanding, preventing, or reducing NSSI among emerging adults with Dark Triad Traits.

## Data Availability

The raw data supporting the conclusions of this article will be made available by the authors, without undue reservation.

## Footnotes

1
www.wjx.cn
