# Supplementary material for: Beyond sum scores: a network analysis of dark triad traits and nonsuicidal self-injury in emerging adults
Source: Front Psychol. 2026 Oct 15;17:1954866. doi: 10.3389/fpsyg.2026.1954866 (PMC13716605; doi:10.3389/fpsyg.2026.1954866)
Supplement: Supplementary file 1 [file Supplementary_file_1.pdf]

Supplementary materials

Table S1. Edge weights in the network model of dark triad traits and NSSI.

	M1	M2	M3	M4	P1	P2	P3	P4	N1	N2	N3	N4	B1	B2	B3	B4	B5	B6	B7	B8	B9	B10	B11	B12
M1		0.205	0.170	0.102	0.091	0.009	0.055	0.000	0.054	0.051	0.044	0.013	0.034	0.000	0.000	0.009	0.000	0.000	0.000	0.000	0.001	-0.001	0.000	-0.014
M2	0.205		0.150	0.108	0.023	0.073	0.057	0.163	0.000	0.000	0.000	0.054	0.000	0.000	0.000	0.000	0.000	0.000	0.000	0.000	0.000	0.000	0.000	0.000
M3	0.170	0.150		0.137	0.140	0.090	0.067	0.000	0.050	0.008	0.000	0.056	0.023	0.000	0.012	0.000	0.000	0.017	0.000	0.000	0.000	0.000	0.000	0.000
M4	0.102	0.108	0.137		0.155	0.096	0.033	0.154	0.000	0.049	0.063	0.000	0.000	0.000	0.000	0.000	0.000	0.000	0.007	0.000	0.000	-0.014	0.000	0.000
P1	0.091	0.023	0.140	0.155		0.213	0.148	0.028	0.071	0.000	0.167	0.072	0.000	0.000	0.000	0.000	0.000	0.000	0.000	0.000	0.000	0.000	0.000	0.000
P2	0.009	0.073	0.090	0.096	0.213		0.182	0.045	0.020	0.000	0.154	0.000	0.026	0.005	0.000	0.031	0.000	0.000	0.000	0.000	0.000	0.000	0.000	0.000
P3	0.055	0.057	0.067	0.033	0.148	0.182		0.087	0.194	0.114	0.001	0.085	0.000	0.000	0.000	0.000	0.000	0.000	0.000	0.000	0.009	0.000	0.000	-0.008
P4	0.000	0.163	0.000	0.154	0.028	0.045	0.087		0.150	0.150	0.099	0.075	0.000	0.000	-0.012	0.000	0.000	0.000	0.000	0.000	0.000	0.000	0.000	0.000
N1	0.054	0.000	0.050	0.000	0.071	0.020	0.194	0.150		0.265	0.137	0.135	0.000	0.000	0.000	0.010	0.000	0.000	0.000	0.000	0.000	-0.003	0.000	0.000
N2	0.051	0.000	0.008	0.049	0.000	0.000	0.114	0.150	0.265		0.138	0.175	0.007	0.000	0.000	0.000	0.000	0.000	0.000	0.000	0.000	-0.009	0.000	0.000
N3	0.044	0.000	0.000	0.063	0.167	0.154	0.001	0.099	0.137	0.138		0.147	0.000	0.000	0.000	0.000	0.000	0.000	0.000	0.001	0.000	0.000	0.000	0.000
N4	0.013	0.054	0.056	0.000	0.072	0.000	0.085	0.075	0.135	0.175	0.147		0.035	0.000	0.009	0.008	0.010	0.000	0.000	0.000	0.023	0.000	0.000	0.000
B1	0.034	0.000	0.023	0.000	0.000	0.026	0.000	0.000	0.000	0.007	0.000	0.035		0.447	0.106	0.109	0.068	0.056	0.000	0.000	0.087	-0.031	0.000	0.000
B2	0.000	0.000	0.000	0.000	0.000	0.005	0.000	0.000	0.000	0.000	0.000	0.000	0.447		0.132	0.078	0.017	0.171	0.000	0.100	0.026	0.000	0.000	0.000
B3	0.000	0.000	0.012	0.000	0.000	0.000	0.000	-0.012	0.000	0.000	0.000	0.009	0.106	0.132		0.245	0.309	0.084	0.080	0.042	0.000	0.000	0.000	0.039
B4	0.009	0.000	0.000	0.000	0.000	0.031	0.000	0.000	0.010	0.000	0.000	0.008	0.109	0.078	0.245		0.184	0.087	0.083	0.014	0.000	0.000	0.000	0.038
B5	0.000	0.000	0.000	0.000	0.000	0.000	0.000	0.000	0.000	0.000	0.000	0.010	0.068	0.017	0.309	0.184		0.119	0.079	0.146	0.013	0.040	0.071	0.004
B6	0.000	0.000	0.017	0.000	0.000	0.000	0.000	0.000	0.000	0.000	0.000	0.000	0.056	0.171	0.084	0.087	0.119		0.184	0.130	0.017	0.014	0.034	0.075
B7	0.000	0.000	0.000	0.007	0.000	0.000	0.000	0.000	0.000	0.000	0.000	0.000	0.000	0.000	0.080	0.083	0.079	0.184		0.087	0.037	0.097	0.093	0.035
B8	0.000	0.000	0.000	0.000	0.000	0.000	0.000	0.000	0.000	0.000	0.001	0.000	0.000	0.100	0.042	0.014	0.146	0.130	0.087		0.234	0.142	0.129	0.039
B9	0.001	0.000	0.000	0.000	0.000	0.000	0.009	0.000	0.000	0.000	0.000	0.023	0.087	0.026	0.000	0.000	0.013	0.017	0.037	0.234		0.263	0.146	0.094
B10	-0.001	0.000	0.000	-0.014	0.000	0.000	0.000	0.000	-0.003	-0.009	0.000	0.000	-0.031	0.000	0.000	0.000	0.040	0.014	0.097	0.142	0.263		0.205	0.306
B11	0.000	0.000	0.000	0.000	0.000	0.000	0.000	0.000	0.000	0.000	0.000	0.000	0.000	0.000	0.000	0.000	0.071	0.034	0.093	0.129	0.146	0.205		0.287
B12	-0.014	0.000	0.000	0.000	0.000	0.000	-0.008	0.000	0.000	0.000	0.000	0.000	0.000	0.000	0.039	0.038	0.004	0.075	0.035	0.039	0.094	0.306	0.287	

Table S2. Bootstrap edge weight estimates with 95% confidence intervals and inclusion frequencies for the Dark Triad–NSSI network.

Edge	Sample weight	Bootstrap mean	CI lower	CI upper	Inclusion frequency
M1--M2	0.205	0.202	0.152	0.252	1.000
M1--M3	0.170	0.168	0.121	0.215	1.000
M2--M3	0.150	0.147	0.095	0.197	1.000
M1--M4	0.102	0.102	0.053	0.152	1.000
M2--M4	0.108	0.107	0.061	0.155	1.000
M3--M4	0.137	0.136	0.089	0.186	1.000
M1--P1	0.091	0.089	0.044	0.138	1.000
M2--P1	0.023	0.025	0.000	0.077	0.815
M3--P1	0.140	0.140	0.091	0.191	1.000
M4--P1	0.155	0.153	0.104	0.198	1.000
M1--P2	0.009	0.017	0.000	0.060	0.693
M2--P2	0.073	0.071	0.022	0.122	0.998
M3--P2	0.090	0.089	0.037	0.141	0.999
M4--P2	0.096	0.095	0.047	0.148	1.000
P1--P2	0.213	0.211	0.155	0.267	1.000
M1--P3	0.055	0.056	0.013	0.099	0.995
M2--P3	0.057	0.055	0.004	0.105	0.979
M3--P3	0.067	0.067	0.016	0.115	0.994
M4--P3	0.033	0.036	0.000	0.086	0.917
P1--P3	0.148	0.145	0.087	0.198	1.000
P2--P3	0.182	0.177	0.126	0.226	1.000
M1--P4	0.000	0.001	0.000	0.013	0.091
M2--P4	0.163	0.158	0.104	0.210	1.000
M3--P4	0.000	0.003	0.000	0.030	0.221
M4--P4	0.154	0.153	0.103	0.202	1.000
P1--P4	0.028	0.030	0.000	0.081	0.878
P2--P4	0.045	0.045	0.000	0.096	0.961
P3--P4	0.087	0.087	0.036	0.137	1.000
M1--N1	0.054	0.053	0.006	0.099	0.988
M2--N1	0.000	0.009	0.000	0.047	0.484
M3--N1	0.050	0.046	0.002	0.091	0.979
M4--N1	0.000	0.001	0.000	0.018	0.106
P1--N1	0.071	0.069	0.017	0.126	0.996
P2--N1	0.020	0.025	0.000	0.071	0.800
P3--N1	0.194	0.190	0.130	0.244	1.000
P4--N1	0.150	0.150	0.098	0.206	1.000
M1--N2	0.051	0.046	0.001	0.095	0.976
M2--N2	0.000	0.007	0.000	0.038	0.398
M3--N2	0.008	0.012	0.000	0.048	0.601
M4--N2	0.049	0.045	0.000	0.095	0.961
P1--N2	0.000	0.010	0.000	0.048	0.494
P2--N2	0.000	0.000	0.000	0.000	0.043
P3--N2	0.114	0.110	0.062	0.158	1.000
P4--N2	0.150	0.149	0.098	0.203	1.000
N1--N2	0.265	0.262	0.206	0.314	1.000
M1--N3	0.044	0.043	0.000	0.091	0.967
M2--N3	0.000	0.000	0.000	0.004	0.047
M3--N3	0.000	0.003	0.000	0.030	0.209
M4--N3	0.063	0.064	0.019	0.109	0.999
P1--N3	0.167	0.164	0.110	0.220	1.000
P2--N3	0.154	0.146	0.095	0.198	1.000
P3--N3	0.001	0.013	0.000	0.060	0.543
P4--N3	0.099	0.099	0.050	0.153	1.000
N1--N3	0.137	0.135	0.080	0.190	1.000
N2--N3	0.138	0.134	0.081	0.184	1.000
M1--N4	0.013	0.019	0.000	0.059	0.734
M2--N4	0.054	0.050	0.004	0.098	0.982
M3--N4	0.056	0.054	0.008	0.101	0.987
M4--N4	0.000	0.002	0.000	0.024	0.169
P1--N4	0.072	0.068	0.017	0.119	0.997
P2--N4	0.000	0.007	0.000	0.038	0.396
P3--N4	0.085	0.085	0.032	0.135	1.000
P4--N4	0.075	0.075	0.026	0.129	0.999
N1--N4	0.135	0.136	0.080	0.190	1.000
N2--N4	0.175	0.173	0.130	0.219	1.000
N3--N4	0.147	0.145	0.089	0.197	1.000
M1--B1	0.034	0.027	0.000	0.057	0.947
M2--B1	0.000	0.004	0.000	0.024	0.391
M3--B1	0.023	0.021	0.000	0.050	0.886
M4--B1	0.000	0.002	0.000	0.018	0.191
P1--B1	0.000	0.003	0.000	0.020	0.309
P2--B1	0.026	0.022	0.000	0.052	0.897
P3--B1	0.000	0.001	0.000	0.011	0.112
P4--B1	0.000	0.000	0.000	0.000	0.021
N1--B1	0.000	0.001	0.000	0.013	0.146
N2--B1	0.007	0.006	0.000	0.028	0.530
N3--B1	0.000	0.000	0.000	0.007	0.087
N4--B1	0.035	0.033	0.003	0.063	0.980
M1--B2	0.000	0.000	0.000	0.000	0.035
M2--B2	0.000	-0.002	-0.018	0.000	0.204
M3--B2	0.000	0.001	0.000	0.012	0.114
M4--B2	0.000	0.001	0.000	0.013	0.173
P1--B2	0.000	0.000	0.000	0.004	0.047
P2--B2	0.005	0.007	0.000	0.029	0.555
P3--B2	0.000	0.000	0.000	0.000	0.023
P4--B2	0.000	0.000	0.000	0.000	0.039
N1--B2	0.000	0.000	0.000	0.001	0.033
N2--B2	0.000	0.001	0.000	0.011	0.147
N3--B2	0.000	0.000	0.000	0.000	0.038
N4--B2	0.000	0.000	0.000	0.000	0.016
B1--B2	0.447	0.443	0.362	0.525	1.000
M1--B3	0.000	0.002	0.000	0.018	0.227
M2--B3	0.000	0.000	-0.006	0.000	0.062
M3--B3	0.012	0.008	0.000	0.032	0.583
M4--B3	0.000	0.002	0.000	0.017	0.187
P1--B3	0.000	0.000	0.000	0.000	0.013
P2--B3	0.000	0.000	0.000	0.008	0.070
P3--B3	0.000	-0.001	-0.011	0.000	0.086
P4--B3	-0.012	-0.008	-0.033	0.000	0.569
N1--B3	0.000	0.000	0.000	0.005	0.053
N2--B3	0.000	0.000	0.000	0.000	0.011
N3--B3	0.000	0.001	0.000	0.011	0.150
N4--B3	0.009	0.007	0.000	0.033	0.525
B1--B3	0.106	0.108	0.017	0.189	0.992
B2--B3	0.132	0.125	0.032	0.225	0.996
M1--B4	0.009	0.007	0.000	0.033	0.522
M2--B4	0.000	0.001	0.000	0.013	0.112
M3--B4	0.000	0.004	0.000	0.030	0.354
M4--B4	0.000	0.001	0.000	0.013	0.115
P1--B4	0.000	0.001	0.000	0.013	0.191
P2--B4	0.031	0.024	0.000	0.050	0.943
P3--B4	0.000	0.001	0.000	0.013	0.144
P4--B4	0.000	0.001	0.000	0.016	0.146
N1--B4	0.010	0.006	0.000	0.025	0.533
N2--B4	0.000	0.002	0.000	0.017	0.201
N3--B4	0.000	0.001	0.000	0.008	0.110
N4--B4	0.008	0.009	0.000	0.040	0.588
B1--B4	0.109	0.110	0.024	0.186	0.998
B2--B4	0.078	0.077	0.000	0.163	0.949
B3--B4	0.245	0.242	0.150	0.335	1.000
M1--B5	0.000	0.000	0.000	0.000	0.020
M2--B5	0.000	0.000	0.000	0.001	0.033
M3--B5	0.000	0.000	0.000	0.000	0.011
M4--B5	0.000	-0.001	-0.015	0.000	0.122
P1--B5	0.000	-0.001	-0.009	0.000	0.097
P2--B5	0.000	0.000	0.000	0.003	0.047
P3--B5	0.000	0.000	0.000	0.000	0.025
P4--B5	0.000	-0.001	-0.014	0.000	0.172
N1--B5	0.000	0.000	0.000	0.002	0.037
N2--B5	0.000	0.001	0.000	0.015	0.172
N3--B5	0.000	-0.001	-0.012	0.000	0.118
N4--B5	0.010	0.010	0.000	0.035	0.675
B1--B5	0.068	0.062	0.000	0.139	0.937
B2--B5	0.017	0.031	0.000	0.105	0.689
B3--B5	0.309	0.304	0.212	0.400	1.000
B4--B5	0.184	0.179	0.091	0.260	1.000
M1--B6	0.000	0.001	0.000	0.015	0.150
M2--B6	0.000	0.002	0.000	0.019	0.211
M3--B6	0.017	0.010	0.000	0.037	0.662
M4--B6	0.000	0.001	0.000	0.013	0.138
P1--B6	0.000	0.000	0.000	0.006	0.089
P2--B6	0.000	0.002	0.000	0.019	0.257
P3--B6	0.000	0.000	0.000	0.000	0.025
P4--B6	0.000	0.000	-0.007	0.000	0.047
N1--B6	0.000	0.002	0.000	0.017	0.293
N2--B6	0.000	0.000	0.000	0.000	0.024
N3--B6	0.000	0.000	0.000	0.000	0.037
N4--B6	0.000	0.002	0.000	0.019	0.214
B1--B6	0.056	0.058	0.000	0.142	0.911
B2--B6	0.171	0.167	0.079	0.266	1.000
B3--B6	0.084	0.084	0.000	0.180	0.966
B4--B6	0.087	0.090	0.003	0.182	0.977
B5--B6	0.119	0.116	0.031	0.202	0.994

Edge	Sample weight	Bootstrap mean	CI lower	CI upper	Inclusion frequency
M1--B7	0.000	0.000	-0.004	0.000	0.041
M2--B7	0.000	0.003	0.000	0.018	0.268
M3--B7	0.000	0.002	0.000	0.016	0.193
M4--B7	0.007	0.004	0.000	0.023	0.348
P1--B7	0.000	0.000	-0.001	0.000	0.037
P2--B7	0.000	0.001	0.000	0.012	0.140
P3--B7	0.000	0.000	0.000	0.003	0.048
P4--B7	0.000	0.000	0.000	0.004	0.063
N1--B7	0.000	0.000	-0.008	0.000	0.071
N2--B7	0.000	-0.001	-0.013	0.000	0.121
N3--B7	0.000	0.001	0.000	0.008	0.077
N4--B7	0.000	0.000	0.000	0.001	0.052
B1--B7	0.000	0.001	0.000	0.018	0.115
B2--B7	0.000	0.001	-0.014	0.026	0.155
B3--B7	0.080	0.080	0.007	0.153	0.985
B4--B7	0.083	0.082	0.007	0.159	0.980
B5--B7	0.079	0.080	0.004	0.159	0.979
B6--B7	0.184	0.181	0.090	0.267	1.000
M1--B8	0.000	-0.001	-0.013	0.000	0.103
M2--B8	0.000	0.000	-0.002	0.000	0.037
M3--B8	0.000	0.003	0.000	0.020	0.328
M4--B8	0.000	0.000	0.000	0.000	0.021
P1--B8	0.000	0.000	0.000	0.000	0.017
P2--B8	0.000	0.001	0.000	0.010	0.119
P3--B8	0.000	0.000	-0.006	0.000	0.064
P4--B8	0.000	-0.001	-0.012	0.000	0.177
N1--B8	0.000	0.000	0.000	0.000	0.019
N2--B8	0.000	-0.001	-0.009	0.000	0.078
N3--B8	0.001	0.001	0.000	0.013	0.213
N4--B8	0.000	0.000	0.000	0.008	0.069
B1--B8	0.000	0.002	0.000	0.028	0.135
B2--B8	0.100	0.092	0.018	0.174	0.987
B3--B8	0.042	0.044	0.000	0.120	0.845
B4--B8	0.014	0.023	0.000	0.086	0.637
B5--B8	0.146	0.139	0.052	0.235	1.000
B6--B8	0.130	0.125	0.034	0.223	0.999
B7--B8	0.087	0.091	0.000	0.179	0.974
M1--B9	0.001	0.004	0.000	0.027	0.337
M2--B9	0.000	0.001	0.000	0.011	0.134
M3--B9	0.000	0.000	0.000	0.004	0.046
M4--B9	0.000	0.000	0.000	0.000	0.041

Edge	Sample weight	Bootstrap mean	CI lower	CI upper	Inclusion frequency
P1--B9	0.000	0.000	0.000	0.000	0.008
P2--B9	0.000	0.000	0.000	0.004	0.050
P3--B9	0.009	0.005	0.000	0.023	0.529
P4--B9	0.000	0.000	0.000	0.005	0.048
N1--B9	0.000	0.001	0.000	0.012	0.177
N2--B9	0.000	0.000	0.000	0.003	0.037
N3--B9	0.000	0.002	0.000	0.015	0.224
N4--B9	0.023	0.021	0.000	0.048	0.917
B1--B9	0.087	0.074	0.003	0.142	0.978
B2--B9	0.026	0.032	0.000	0.107	0.707
B3--B9	0.000	-0.002	-0.038	0.005	0.129
B4--B9	0.000	0.002	0.000	0.034	0.165
B5--B9	0.013	0.022	0.000	0.092	0.573
B6--B9	0.017	0.030	0.000	0.111	0.643
B7--B9	0.037	0.036	0.000	0.115	0.789
B8--B9	0.234	0.229	0.128	0.333	1.000
M1--B10	-0.001	-0.004	-0.026	0.000	0.380
M2--B10	0.000	-0.001	-0.013	0.000	0.185
M3--B10	0.000	-0.001	-0.015	0.000	0.179
M4--B10	-0.014	-0.009	-0.032	0.000	0.665
P1--B10	0.000	-0.002	-0.013	0.000	0.221
P2--B10	0.000	-0.002	-0.016	0.000	0.187
P3--B10	0.000	0.000	0.000	0.000	0.010
P4--B10	0.000	-0.002	-0.017	0.000	0.246
N1--B10	-0.003	-0.003	-0.021	0.000	0.339
N2--B10	-0.009	-0.007	-0.027	0.000	0.540
N3--B10	0.000	0.000	0.000	0.001	0.032
N4--B10	0.000	0.000	0.000	0.000	0.022
B1--B10	-0.031	-0.019	-0.062	0.000	0.651
B2--B10	0.000	-0.001	-0.016	0.000	0.069
B3--B10	0.000	0.009	0.000	0.049	0.415
B4--B10	0.000	0.000	0.000	0.000	0.017
B5--B10	0.040	0.031	0.000	0.088	0.826
B6--B10	0.014	0.019	0.000	0.080	0.611
B7--B10	0.097	0.092	0.016	0.165	0.994
B8--B10	0.142	0.140	0.042	0.246	0.997
B9--B10	0.263	0.256	0.156	0.359	1.000
M1--B11	0.000	-0.001	-0.012	0.000	0.100
M2--B11	0.000	0.000	-0.008	0.000	0.075
M3--B11	0.000	0.003	0.000	0.018	0.304
M4--B11	0.000	0.000	0.000	0.006	0.058

Edge	Sample weight	Bootstrap mean	CI lower	CI upper	Inclusion frequency
P1--B11	0.000	0.000	-0.001	0.000	0.033
P2--B11	0.000	0.000	0.000	0.003	0.045
P3--B11	0.000	0.000	-0.005	0.000	0.069
P4--B11	0.000	-0.001	-0.010	0.000	0.139
N1--B11	0.000	0.000	0.000	0.004	0.056
N2--B11	0.000	0.000	0.000	0.000	0.029
N3--B11	0.000	0.000	0.000	0.002	0.044
N4--B11	0.000	0.000	-0.005	0.000	0.045
B1--B11	0.000	0.003	0.000	0.032	0.163
B2--B11	0.000	0.001	-0.005	0.021	0.123
B3--B11	0.000	0.009	0.000	0.055	0.405
B4--B11	0.000	-0.003	-0.036	0.000	0.126
B5--B11	0.071	0.066	0.000	0.144	0.949
B6--B11	0.034	0.034	0.000	0.104	0.785
B7--B11	0.093	0.090	0.012	0.171	0.985
B8--B11	0.129	0.126	0.025	0.231	0.996
B9--B11	0.146	0.142	0.048	0.242	0.999
B10--B11	0.205	0.207	0.094	0.321	1.000
M1--B12	-0.014	-0.007	-0.030	0.000	0.540
M2--B12	0.000	-0.002	-0.020	0.000	0.258
M3--B12	0.000	0.000	0.000	0.000	0.023
M4--B12	0.000	-0.004	-0.025	0.000	0.367
P1--B12	0.000	0.000	-0.005	0.000	0.064
P2--B12	0.000	0.000	0.000	0.000	0.019
P3--B12	-0.008	-0.004	-0.022	0.000	0.438
P4--B12	0.000	-0.001	-0.010	0.000	0.107
N1--B12	0.000	-0.002	-0.015	0.000	0.256
N2--B12	0.000	-0.002	-0.016	0.000	0.194
N3--B12	0.000	0.002	0.000	0.017	0.199
N4--B12	0.000	-0.001	-0.015	0.000	0.151
B1--B12	0.000	-0.001	-0.014	0.000	0.074
B2--B12	0.000	0.002	0.000	0.023	0.165
B3--B12	0.039	0.030	0.000	0.083	0.823
B4--B12	0.038	0.034	0.000	0.092	0.872
B5--B12	0.004	0.015	0.000	0.070	0.553
B6--B12	0.075	0.065	0.000	0.144	0.954
B7--B12	0.035	0.041	0.000	0.122	0.821
B8--B12	0.039	0.043	0.000	0.126	0.791
B9--B12	0.094	0.093	0.008	0.180	0.983
B10--B12	0.306	0.304	0.193	0.425	1.000
B11--B12	0.287	0.285	0.169	0.394	1.000

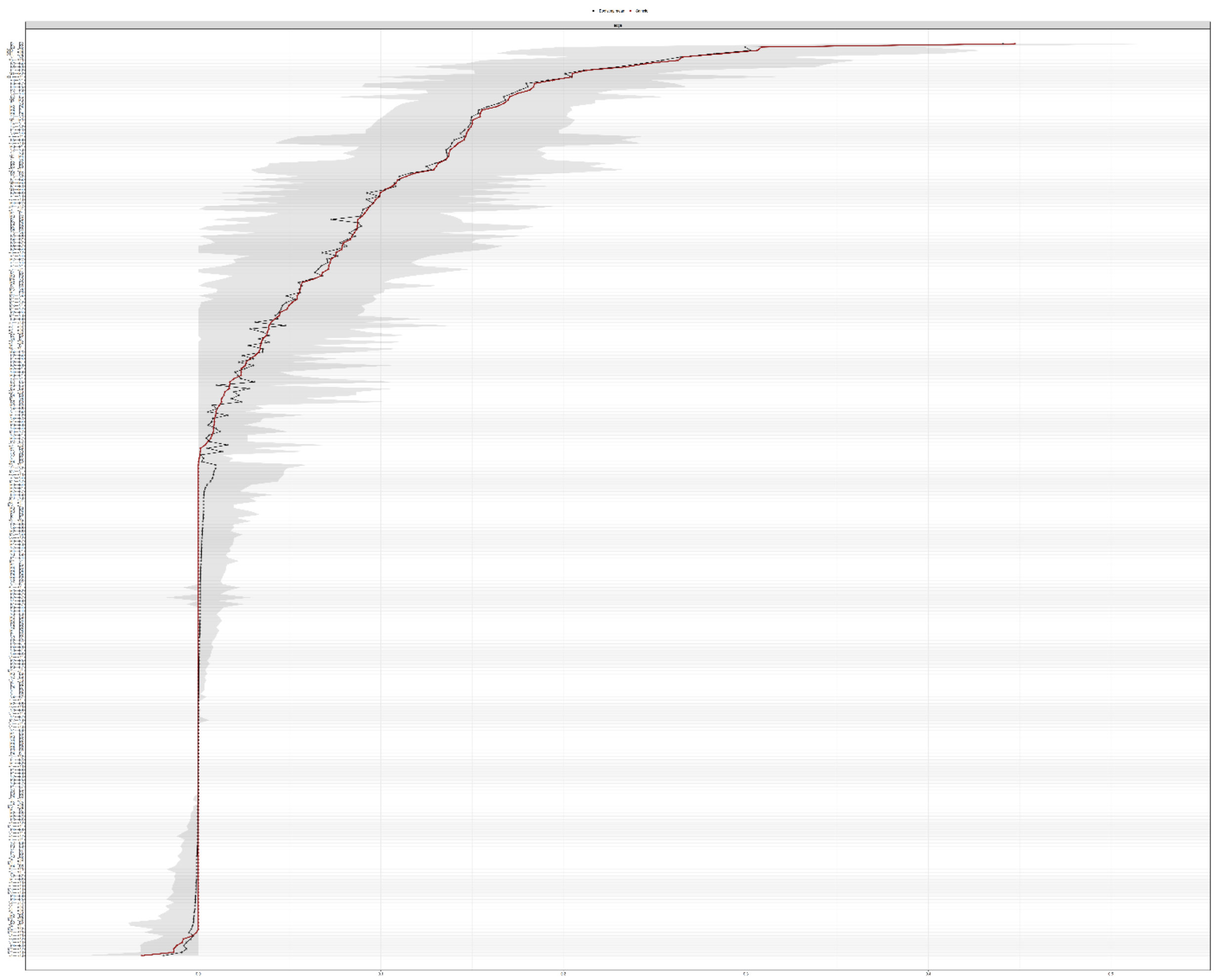

Supplementary Figure S1. Accuracy of edge weights in the dark triad traits and NSSI network.

Note: The red line depicts the sample edge weights and the gray bar depicts the bootstrapped confidence interval.

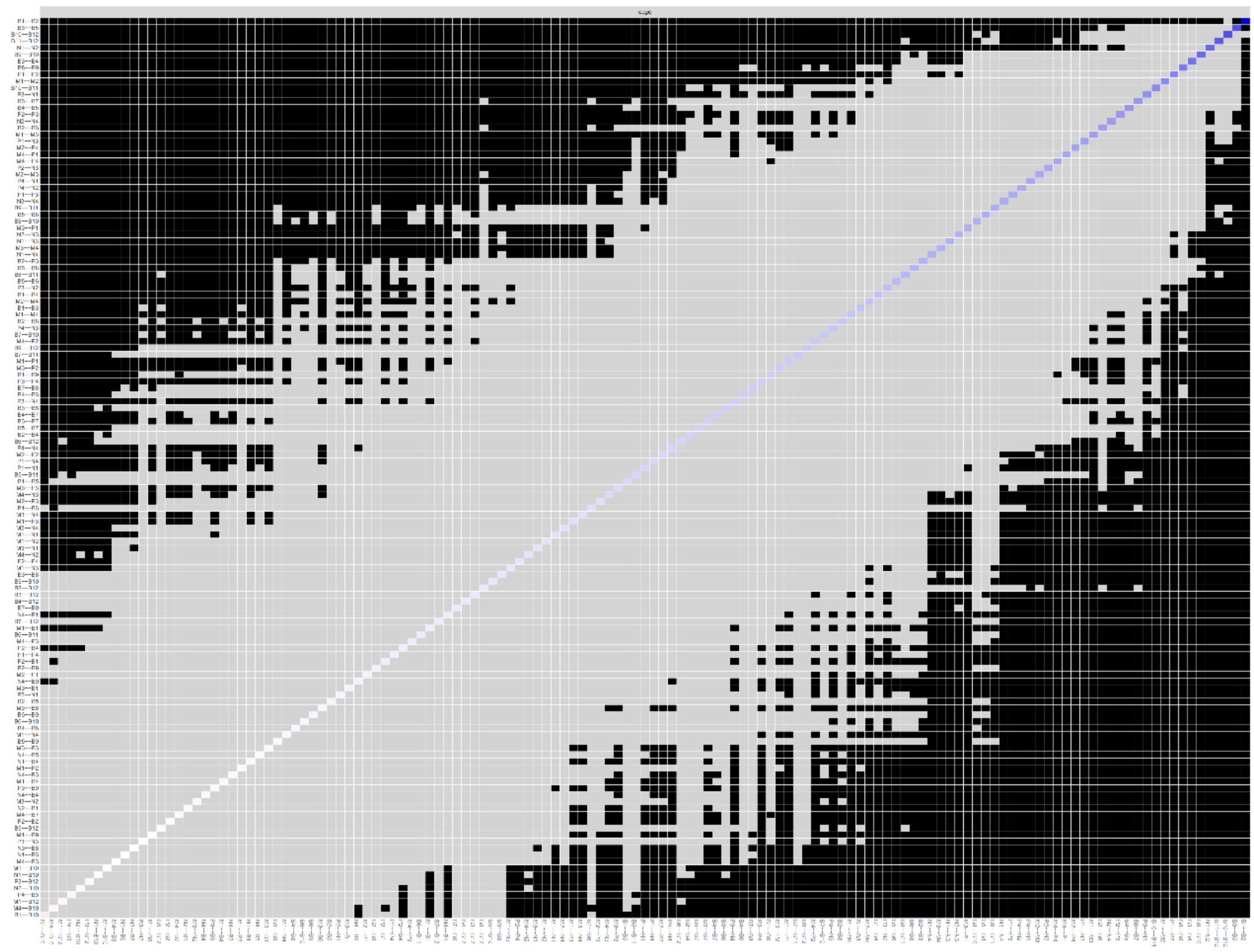

Supplementary Figure S2. Bootstrapped difference test for edge weights in the dark triad traits and NSSI network.

Note: Gray boxes indicate edge weights that do not differ significantly from one another, while black boxes indicate edge weights that do differ significantly. Blue and red boxes on the diagonal correspond to edge weights with positive and negative correlations, respectively.

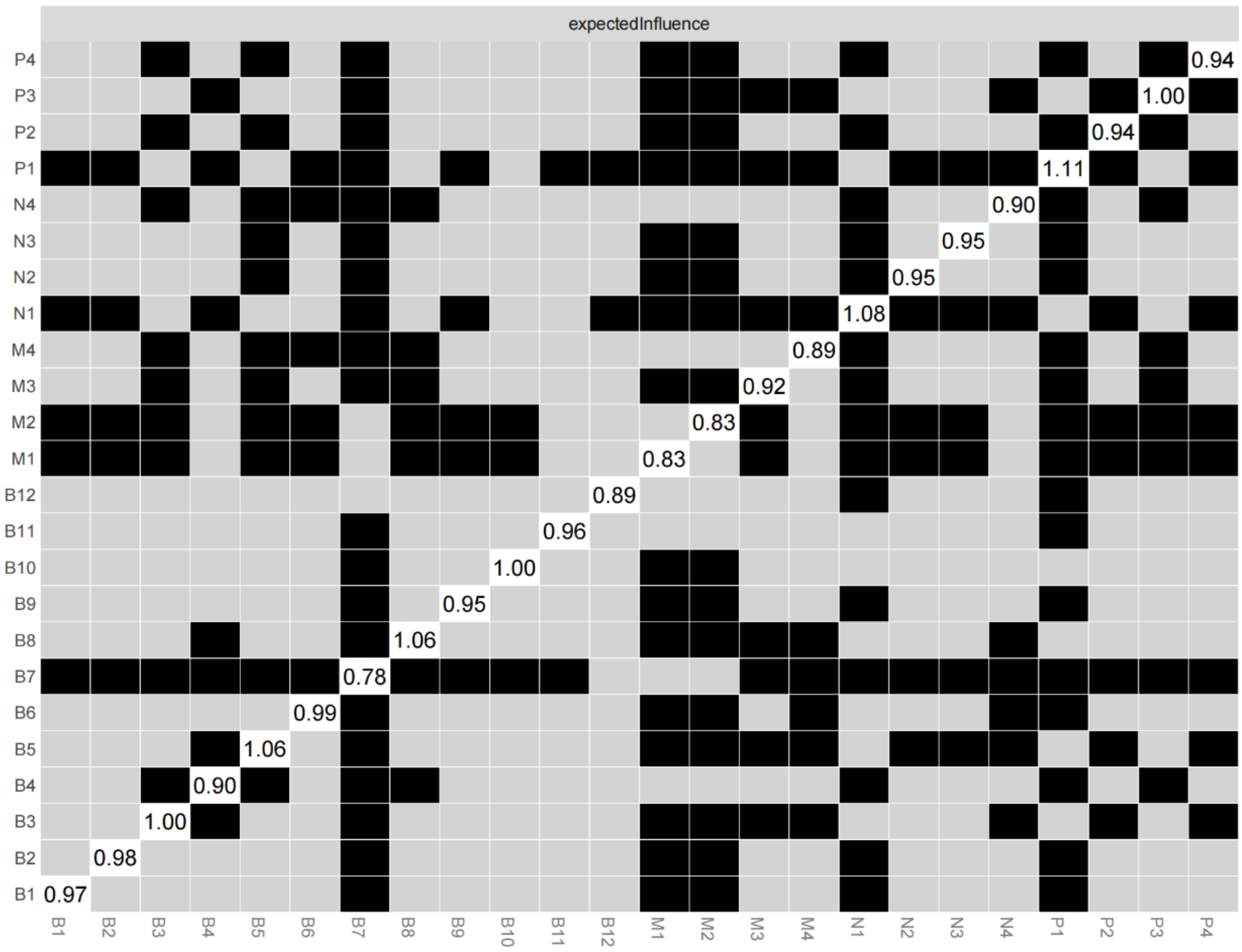

Supplementary Figure S3. Bootstrapped difference test for node expected influences in the dark triad traits and NSSI network.

Note: Gray boxes indicate node expected influences that do not differ significantly from one another, while black boxes indicate node expected influences that do differ significantly. The numbers in the white boxes (i.e., diagonal line) represent the values of node expected influences.

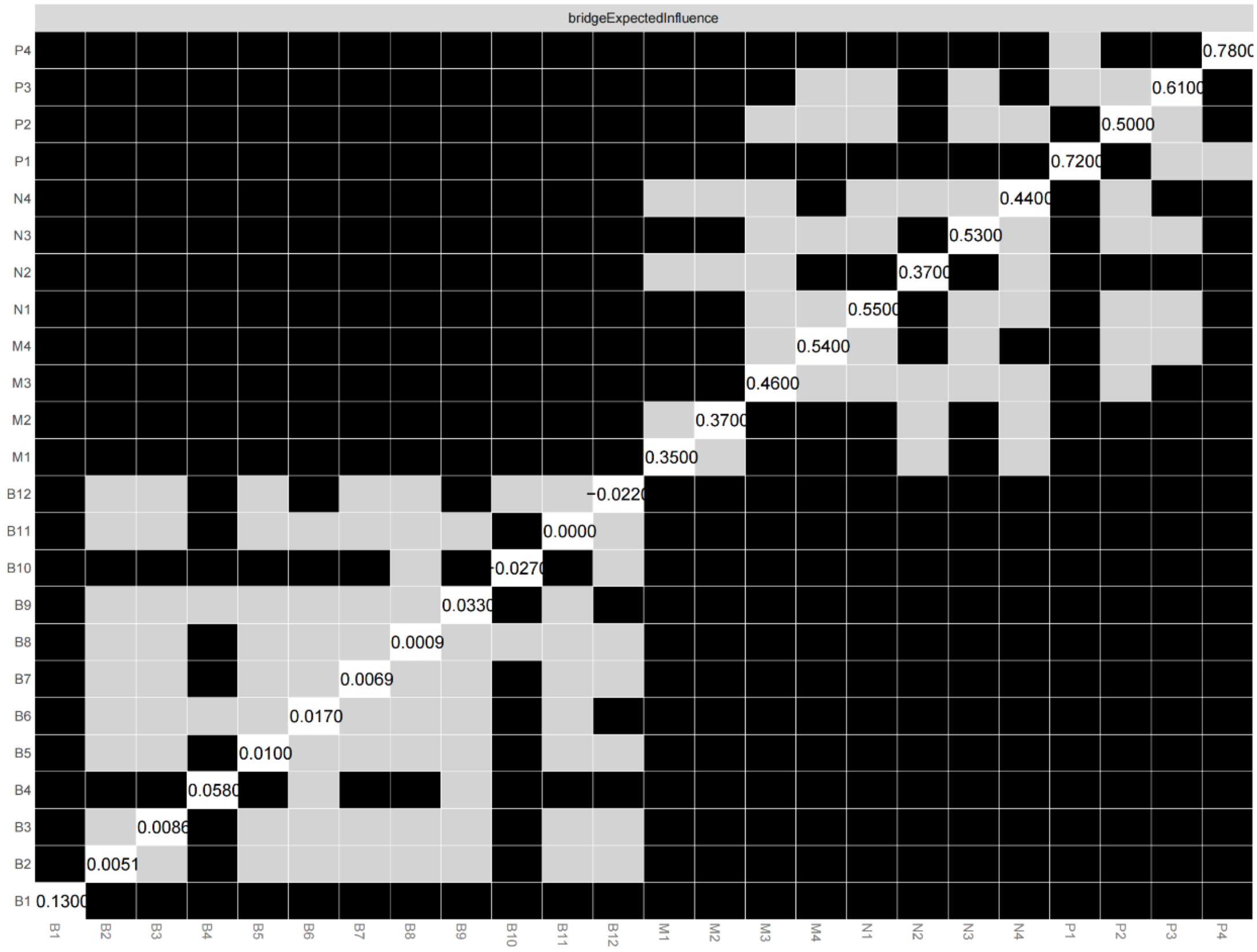

Supplementary Figure S4. Bootstrapped difference test for node bridge expected influences in the dark triad traits and NSSI network.

Note: Gray boxes indicate node bridge expected influences that do not differ significantly from one another, while black boxes indicate node bridge expected influences that do differ significantly. The numbers in the white boxes (i.e., diagonal line) represent the values of node bridge expected influences.
